# Loss of the glycosyltransferase Galnt11 affects vitamin D homeostasis and bone composition

**DOI:** 10.1016/j.jbc.2024.107164

**Published:** 2024-03-12

**Authors:** E. Tian, Caroline Rothermel, Zachary Michel, Luis Fernandez de Castro, Jeeyoung Lee, Tina Kilts, Tristan Kent, Michael T. Collins, Kelly G. Ten Hagen

**Affiliations:** 1 Developmental Glycobiology Section, National Institute of Dental and Craniofacial Research, National Institutes of Health, Bethesda, Maryland, USA; 2 Skeletal Disorders and Mineral Homeostasis Section, National Institute of Dental and Craniofacial Research, National Institutes of Health, Bethesda, Maryland, USA

**Keywords:** Galnt11, O-glycosylation, megalin, vitamin D binding protein, bone remodeling, cortical bone, trabecular bone, vitamin D, calcium, parathyroid hormone, kidney

## Abstract

O-glycosylation is a conserved posttranslational modification that impacts many aspects of organismal viability and function. Recent studies examining the glycosyltransferase Galnt11 demonstrated that it glycosylates the endocytic receptor megalin in the kidneys, enabling proper binding and reabsorption of ligands, including vitamin D–binding protein (DBP). *Galnt11*-deficient mice were unable to properly reabsorb DBP from the urine. Vitamin D plays an essential role in mineral homeostasis and its deficiency is associated with bone diseases such as rickets, osteomalacia, and osteoporosis. We therefore set out to examine the effects of the loss of *Galnt11* on vitamin D homeostasis and bone composition. We found significantly decreased levels of serum 25-hydroxyvitamin D and 1,25-dihydroxyvitamin D, consistent with decreased reabsorption of DBP. This was accompanied by a significant reduction in blood calcium levels and a physiologic increase in parathyroid hormone (PTH) in *Galnt11*-deficient mice. Bones in *Galnt11*-deficient mice were smaller and displayed a decrease in cortical bone accompanied by an increase in trabecular bone and an increase in a marker of bone formation, consistent with PTH-mediated effects on bone. These results support a unified model for the role of Galnt11 in bone and mineral homeostasis, wherein loss of Galnt11 leads to decreased reabsorption of DBP by megalin, resulting in a cascade of disrupted mineral and bone homeostasis including decreased circulating vitamin D and calcium levels, a physiological increase in PTH, an overall loss of cortical bone, and an increase in trabecular bone. Our study elucidates how defects in O-glycosylation can influence vitamin D and mineral homeostasis and the integrity of the skeletal system.

Mucin-type O-linked glycosylation is an essential posttranslational modification that has diverse roles in many aspects of biology (1, 2). This modification is initiated by a large family of enzymes (20 members in mammals and 10 in *Drosophila*) known as the UDP-N-acetylgalactosamine:polypeptide N-acetylgalactosaminyltransferases (GALNTs), which catalyze the transfer of the sugar group GalNAc onto serine and threonine residues of membrane-bound and secreted proteins (1, 2). Enzymatic studies have demonstrated that members of this family have overlapping, yet unique substrate specificities as well as unique patterns of expression (1, 2, 3, 4, 5, 6, 7). Studies in model organisms have shown that certain family members are essential for viability and others are involved in diverse biological processes including cell adhesion, protein processing, secretion, and organ development and function (2, 8, 9, 10, 11, 12, 13, 14, 15). Mutations in human *GALNT3* are responsible for the disease hyperphosphatemic familial tumoral calcinosis (16), and a recent study has identified mutations in *GALNT2* that result in a novel congenital disorder of glycosylation (17). Taken together, these studies collectively highlight the importance of O-linked glycosylation and this enzyme family in health and disease.

Genome-wide association studies identified one member of this family, *GALNT11*, as being associated with kidney functional decline in humans (18). Indeed, *GALNT11* is highly expressed in the kidney, being the most abundant family member within this organ (19). In support of a role for *GALNT11* in the kidney, mice deficient for *Galnt11* also displayed kidney dysfunction (19). Mechanistically, it was found that Galnt11 normally glycosylates the endocytic receptor megalin in the kidney. Proper glycosylation of megalin is required for its ability to bind and reabsorb ligands. Mice deficient for *Galnt11* displayed reduced reabsorption of multiple megalin ligands from the glomerular filtrate, including retinol binding protein, albumin, α-1-microglobulin, and vitamin D–binding protein (DBP), resulting in low molecular weight proteinuria (19).

Megalin is an essential endocytic receptor in the proximal tubules of the kidney that is responsible for binding and endocytosing multiple ligands, including DBP, thereby reabsorbing 25-hydroxyvitamin D from the glomerular filtrate into renal epithelial cells (20). Within renal epithelial cells, 25-hydroxyvitamin D is converted to its active form, 1,25-dihydroxyvitamin D by the 1α-hydroxylase. The hormonally active form of vitamin D stimulates the intestinal absorption of calcium and thereby is a major regulator of bone mineral homeostasis (21). The role of vitamin D in bone health is well characterized, and its deficiency is associated with bone diseases such as osteoporosis, osteomalacia, and rickets (22).

The association between poor glomerular vitamin D reabsorption and bone disease has been previously characterized and is described as part of the phenomenon of renal osteodystrophy (23). A major complication of chronic kidney disease, renal osteodystrophy includes skeletal abnormalities caused by decreased levels of circulating 1,25-dihydroxyvitamin D and altered calcium-phosphate homeostasis. As the maintenance of appropriate levels of calcium is essential for the regulation of many physiological processes, the parathyroid gland responds to low circulating calcium levels by releasing parathyroid hormone (PTH), which stimulates bone remodeling.

Bone remodeling has differential effects on cortical bone and trabecular bone; PTH has been observed to have an anabolic effect on trabecular bone but a catabolic effect on cortical bone (24). In this instance, the endocortical surface of cortical bone undergoes osteoclastic bone resorption and becomes increasingly porous, resembling trabecular bone. The observation of increased trabecular bone and decreased cortical bone in renal osteodystrophy patients has been attributed to chronically elevated levels of PTH, resulting from decreased circulating calcium levels caused by decreased vitamin D reabsorption within the kidneys.

Given our previous work documenting that Galnt11 affects megalin stability and ligand-binding properties (19), we set out to examine the effects of *Galnt11* deficiency on vitamin D homeostasis and bone health. Here we find that loss of *Galnt11* resulted in decreased circulating levels of vitamin D and calcium, with a compensatory increase in circulating PTH levels. Consistent with increased PTH levels, we observe noteworthy trends toward decreased cortical bone and increased trabecular bone in *Galnt11*-deficient animals. Our results are consistent with the loss of *Galnt11* affecting vitamin D homeostasis, calcium levels, PTH levels, and bone composition through its effects on megalin in the kidney. These results may provide insight into the effects of *GALNT11* mutations on skeletal homeostasis in humans.

## Results

### *Galnt11*-deficient mice have decreased circulating vitamin D, calcium, and increased PTH

**Figure 1:**
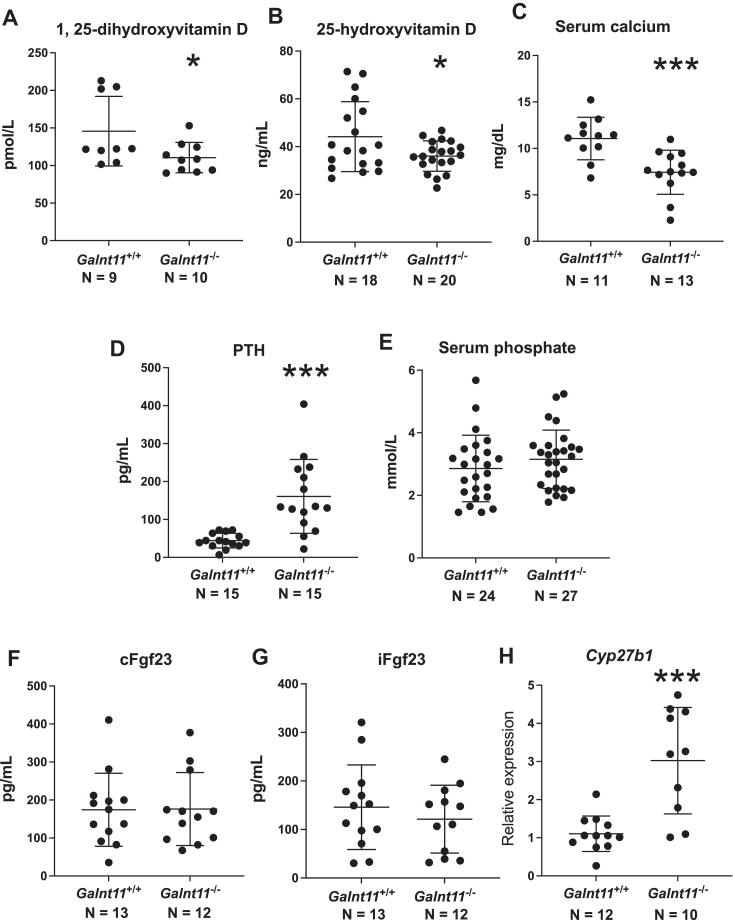
***Galnt11*-deficient mice have altered blood chemistry.***Galnt11*-deficient mice (*Galnt11*^−/−^) showed a significant decrease in circulating 1,25-dihydroxyvitamin D levels (*A*) and 25-hydroxyvitamin D levels (*B*) relative to *Galnt11*^+/+^ (*WT*) littermate controls. *C*, *Galnt11*-deficient mice showed a significant decrease in circulating calcium levels relative to *Galnt11*^+/+^ littermate controls. *D*, *Galnt11*-deficient mice displayed a significant increase in serum parathyroid hormone (PTH) levels relative to *Galnt11*^+/+^ littermate controls. *E*, no significant difference in serum phosphate levels between *Galnt11*^+/+^ and *Galnt11*^−/−^ mice was observed. No significant differences were observed between *WT* and *Galnt11*^−/−^ littermates in levels of inactive, cleaved Fgf23 (cFgf23) (*F*) or active, intact Fgf23 (iFgf23) (*G*). *H*, QPCR analysis of gene expression of *Cyp27b1* shows a significant increase in *Galnt11*^−/−^ kidneys relative to *WT.* N = number of mice analyzed. Significance values are shown in each graph. ∗*p* < 0.05; ∗∗∗*p* < 0.001.

We previously reported that loss of Galnt11-mediated glycosylation of megalin resulted in reduced reabsorption of DBP (19). To interrogate the direct effects of decreased DBP reabsorption, we first began by examining the levels of the active and inactive forms of vitamin D, 1,25-dihydroxyvitamin D and 25-hydroxyvitamin D, respectively. Blood chemistry showed significant decreases in 1,25-dihydroxyvitamin D and 25-hydroxyvitamin D in *Galnt11*-deficient mice relative to *WT* littermates (Fig. 1, *A* and *B*), in line with defects in megalin-mediated reabsorption of DBP. When the data for males and females were analyzed separately, there was a trend toward lower 1,25-dihydroxyvitamin D and 25-hydroxyvitamin D in males and a significant decrease in 25-hydroxyvitamin D with a trend toward lower 1,25-dihydroxyvitamin D in females (Figs. S1 and S2).

Given that the active form of vitamin D stimulates the intestinal reabsorption of calcium, we hypothesized that blood calcium levels may be altered in the *Galnt11*-deficient mice. As predicted, blood calcium levels were significantly decreased in *Galnt11*-deficient mice relative to *WT* littermates (Figs. 1*C*, S1, and S2). Given that low blood calcium levels can trigger an increase in the bone remodeling hormone PTH and given that PTH is cleared from the circulation by megalin (25), we next examined levels of PTH. Consistent with low blood calcium levels, we found a highly significant increase in PTH in *Galnt11*-deficient mice relative to *WT* controls (Fig. 1*D*). The differences in PTH levels remained highly significant when males and females were examined separately (Figs. S1 and S2). No differences in blood phosphate were noted (Figs. 1*E*, S1, and S2). Likewise, there were no differences in the levels of intact (active) or cleaved (inactive) Fgf23, the phosphate regulating hormone produced by osteocytes (Figs. 1, *F* and *G*, S1, and S2), suggesting that phosphate regulation remains intact in *Galnt11*-deficient animals. Taken together, loss of *Galnt11*-mediated glycosylation of megalin affects circulating active and inactive vitamin D levels, calcium levels, and PTH levels.

Low calcium and increased PTH levels are expected to increase the expression of the gene encoding the 1α-hydroxylase, which converts 25-hydroxyvitamin D to 1,25-dihydroxyvitamin D in the proximal tubules of the kidney (26). We therefore examined the expression of *Cyp27b1* within the kidneys of *WT* and *Galnt11*-deficient mice. Consistent with the role of altered calcium and PTH levels, we found a significant increase in the expression of *Cyp27b1* in *Galnt11*-deficient kidneys (Figs. 1*H*, S1, and S2). However, since the effects of the 1α-hydroxylase enzyme would be dependent on the proper reabsorption of 25-hydroxyvitamin D into the cells of kidney, levels of 1,25-dihydroxyvitamin D may be expected to remain low in *Galnt11*-deficient animals, as is observed.

### *Galnt11*-deficient mice have an altered skeletal phenotype

**Figure 2:**
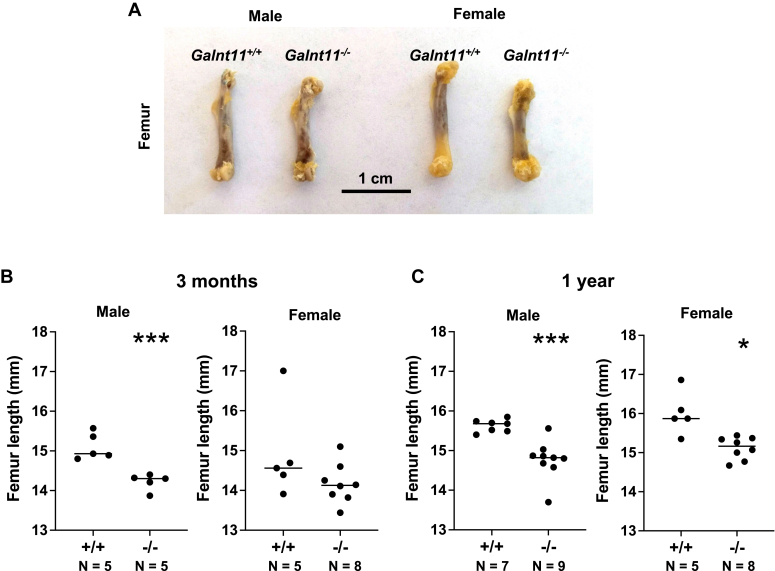
***Galnt11*-deficient mice display short femurs.***A*, morphological overview of femurs from male and female, *Galnt11*^+/+^ and *Galnt11*^−/−^ mice. *B*, male *Galnt11*^−/−^ mice showed a significant decrease and females showed a trend toward a decrease in femur length compared to *Galnt11*^+/+^ littermates at 3 months of age. *C*, both male and female *Galnt11*^−/−^ mice showed a significant decrease in femur length compared to *Galnt11*^+/+^ littermates at 1 year of age. Scale bar represents 1 cm. ∗*p* < 0.05; ∗∗∗*p* < 0.001. N = number of mice analyzed.

Given the changes noted in circulating vitamin D, calcium, and PTH, we next examined the bones of *Galnt11*-deficient mice. We began by measuring the overall length of femurs from *WT* and *Galnt11*-deficient mice. As shown in Figure 2, *A* and *C*, both male and female *Galnt11*-deficient mice displayed significantly reduced femur length compared to *WT* at 1 year of age. Male *Galnt11*-deficient mice displayed significantly reduced femur length and females had a trend toward reduced length at 3 months of age relative to *WT* (Fig. 2*B*).

**Figure 3:**
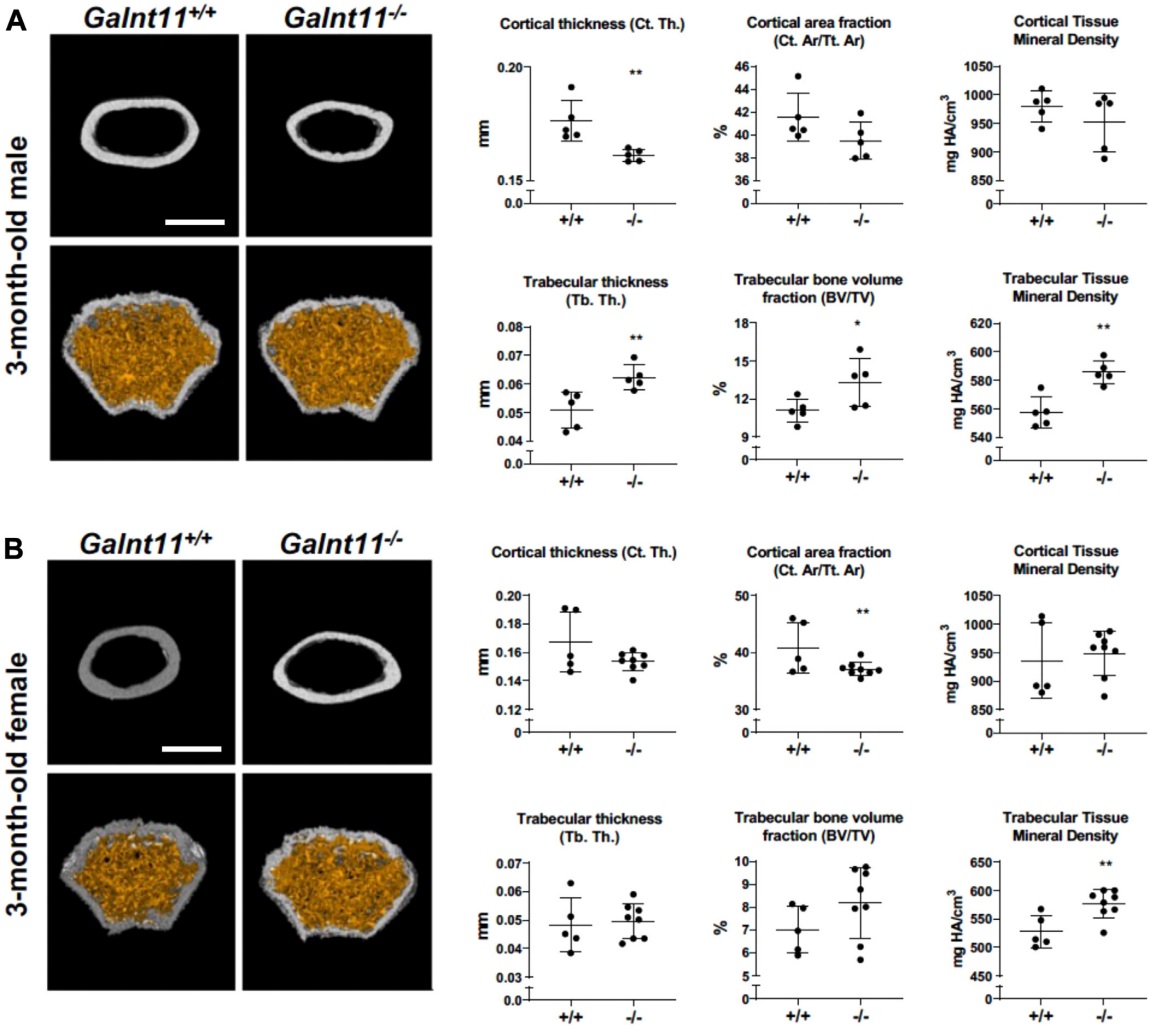
**Altered bone composition in *Galnt11*-deficient mice.** Microcomputed tomography of the femurs from three-month-old *Galnt11*^+/+^ and *Galnt11*^−/−^ mice are shown. Three-dimensional reconstructions of cortical bone displayed in *white* (*top panel*) and trabecular bone displayed in *orange* (*bottom panel*) for males (*A*) and females (*B*) are shown. Axial view of the midshaft region of cortical bone (*top*) and axial view of the distal diaphyseal region of trabecular bone (*bottom*) are shown. Quantitative morphometric and bone mineral density analysis of femurs from three-month-old male *Galnt11*^+/+^ (n = 5) and *Galnt11*^−/−^ (n = 5) mice is shown. Male *Galnt11*^−/−^ mice exhibited a statistically significant decrease in cortical thickness (*p* = 0.007), a statistically significant increase in trabecular thickness (*p* = 0.01), and statistically significant increases in trabecular bone volume fraction (*p* = 0.048) and trabecular tissue mineral density (*p* = 0.0015) relative to *Galnt11*^+/+^ mice. Quantitative morphometric and bone mineral density analysis of femurs from three-month-old female *Galnt11*^+/+^ (n = 5) and *Galnt11*^−/−^ mice (n = 8). Female *Galnt11*^−/−^ mice exhibited a statistically significant decrease in cortical area fraction (*p* = 0.04) and a statistically significant increase in trabecular tissue mineral density (*p* = 0.007) relative to *Galnt11*^+/+^ mice. Results are shown as mean ± SD. Scale bar represents 1 mm. ∗*p* < 0.05; ∗∗*p* < 0.01. BV/TV, trabecular bone volume fraction; Ct. Th, thickness of the cortex; Ct.Ar/Tt.Ar, cortical area fraction; Tb. Th, trabecular bone thickness.

We next performed additional analyses of the femurs of *WT versus Galnt11*-deficient mice. Von Kossa staining, which detects calcium deposition in bones, revealed no significant difference between *WT* and *Galnt11*-deficient mice (data not shown), indicating the absence of gross mineralization abnormalities (osteomalacia). We then performed quantitative microcomputed tomography (micro-CT) to analyze cortical and trabecular bone. Shown in Figure 3 are representative images along with the quantitation of micro-CT measurements, including the following: cortical thickness; trabecular thickness; cortical area fraction; trabecular bone volume fraction; cortical tissue mineral density; and trabecular tissue mineral density. Figure 3 summarizes the data from 3-month-old male and female *WT* and *Galnt11*-deficient mice. Male *Galnt11*-deficient mice exhibited a statistically significant decrease in cortical thickness and a statistically significant increase in trabecular thickness relative to *WT* littermate controls (Fig. 3*A*). Additionally, male *Galnt11*-deficient mice displayed a statistically significant increase in trabecular bone volume fraction and trabecular tissue mineral density relative to *WT* (Fig. 3*A*). In female mice, there was a trend toward decreased cortical thickness along with a statistically significant decrease in cortical area fraction relative to *WT* littermate controls (Fig. 3*B*). There was also a trend toward increased trabecular bone volume fraction and a statistically significant increase in trabecular tissue mineral density, similar to what was observed in males (Fig. 3*B*). These results are consistent with a decrease in cortical bone volume and an apparent increase in trabecular tissue that would be expected in instances of elevated levels of PTH.

**Figure 4:**
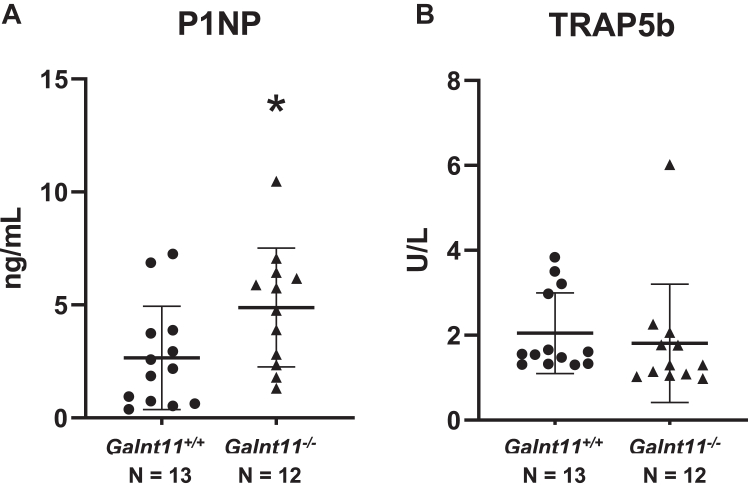
**Circulating bone turnover markers in *WT* and *Galnt11-deficient* mice.***A*, assays to detect procollagen type I intact N-terminal propeptide (P1NP), a marker of bone formation showed a significant increase in *Galnt11*^−/−^ mice relative to *WT* controls. *B*, tartrate-resistant acid phosphatase (TRAP5b), a marker of bone resorption, showed no significant differences between *Galnt11*^−/−^ mice and *WT* controls. Significance values are shown in each graph. ∗*p* < 0.05; N = number of mice analyzed.

To gain more insight into the specific effects of the loss of *Galnt11* on bone, we next examined markers of bone formation (procollagen type I intact N-terminal propeptide; P1NP) and bone resorption (tartrate-resistant acid phosphatase; TRAP5b). As shown in Figure 4*A*, the levels of P1NP were significantly increased in *Galnt11*-deficient mice. When examining male and females separately, there were trends toward an increase in P1NP, although significance was not reached (Figs. S1 and S2). When examining TRAP5b, a marker of bone resorption, no significant differences were seen in either gender or among total animals examined (Figs. 4*B*, S1, and S2).

**Figure 5:**
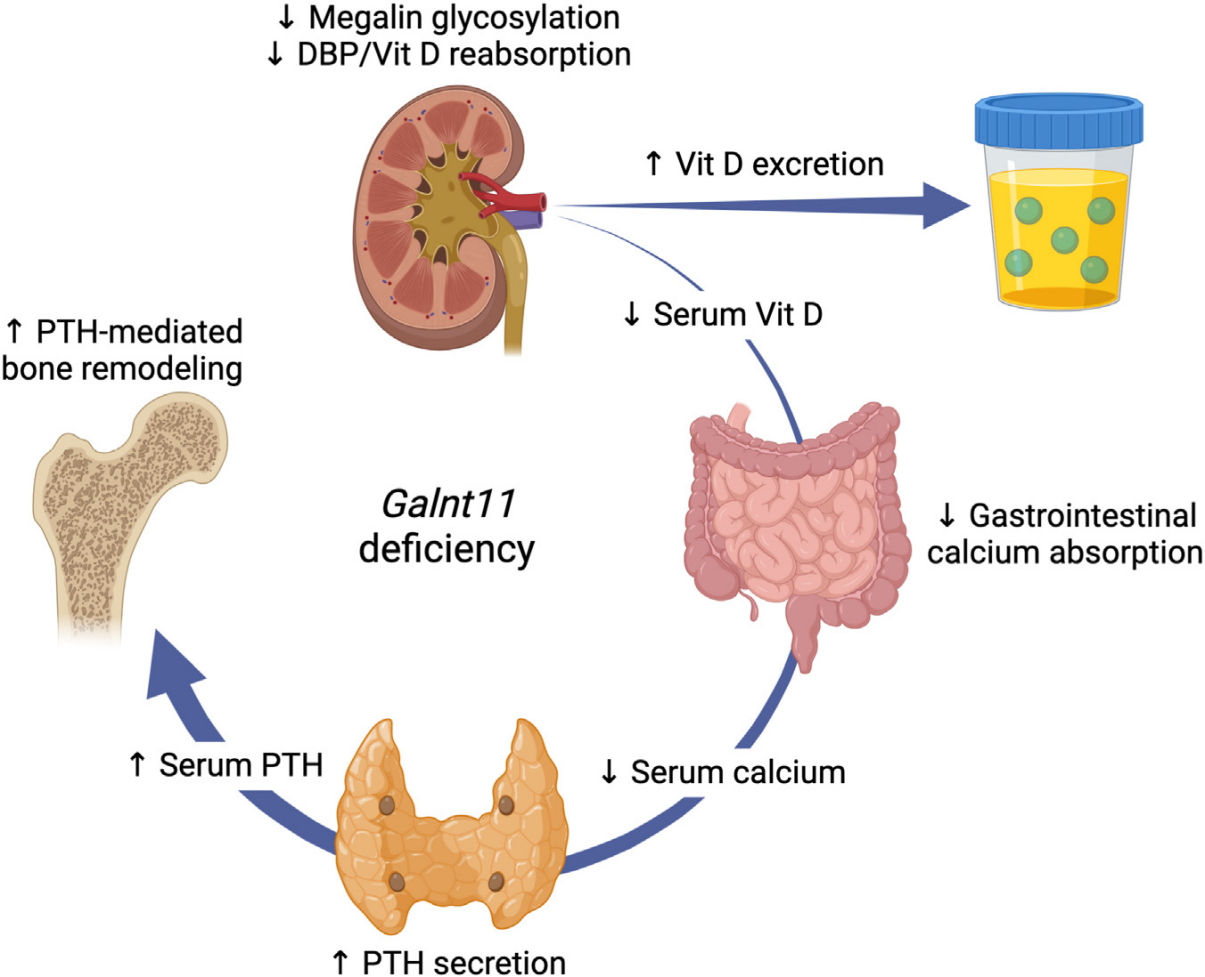
**Summary of vitamin, mineral, and bone changes in *Galnt11*-deficient mice.** Loss of *Galnt11* leads to decreased glycosylation of the endocytic receptor megalin, which affects its ability to reabsorb ligands, including DBP. Decreased DBP reabsorption results in decreased serum vitamin D levels, which then triggers decreased gastrointestinal calcium absorption, leading to decreased serum calcium levels. Low serum calcium triggers the secretion of PTH resulting in increased serum PTH levels. PTH mediates bone remodeling, resulting in an overall loss of cortical bone and an increase in trabecular bone. DBP, vitamin D–binding protein; PTH, parathyroid hormone.

Taken together, our study documents the downstream consequences of altered megalin glycosylation by *Galnt11.* Hypoglycosylated megalin, which fails to properly bind and reabsorb ligands, including DBP, results in decreased circulating vitamin D levels. Consistent with decreased vitamin D levels, we observe decreased levels of blood calcium, which likely contributes to increased levels of PTH and bone remodeling. We demonstrate a unified, physiologically mediated cascade of perturbations that result from altered megalin function, supporting a role for Galnt11-mediated glycosylation of megalin in mineral and bone homeostasis (Fig. 5).

## Discussion

Genome-wide association studies have previously reported an association between *GALNT11* and chronic kidney disease (18). In our previous work, we demonstrated that loss of Galnt11 in the kidney resulted in loss of glycosylation of the endocytic receptor megalin, which affected the ability of megalin to reabsorb many proteins, including DBP (19). Reduced reabsorption of DBP would be expected to impact circulating levels of vitamin D, which prompted the current study to address this question directly. Indeed, circulating levels of both 1,25-dihydroxyvitamin D and 25-hydroxyvitamin D, both of which are bound by DBP, were significantly decreased in *Galnt11*-deficient mice relative to *WT* littermate controls. As 1,25-dihydroxyvitamin D stimulates intestinal reabsorption of calcium, we investigated whether calcium levels were affected. Indeed, circulating calcium levels were also significantly decreased in *Galnt11*-deficient animals relative to *WT* littermate controls. Consistent with the anticipated physiological response to low circulating calcium levels, we also found significantly elevated levels of PTH in the blood of *Galnt11*-deficient mice. Finally, the secondary hyperparathyroidism induced by low blood calcium levels resulted in alterations in skeletal integrity. Taken together, our results support a model wherein the direct effects of Galnt11 on megalin influence its ability to bind DBP, impacting vitamin D reabsorption (Fig. 5).

Analysis of the bones of *Galnt11*-deficient mice revealed a noteworthy trend of decreased cortical bone and increased trabecular bone, consistent with the effects of elevated PTH. While statistical significance varied across these subgroups, there were clear instances of significantly decreased cortical thickness or area and increased trabecular thickness. Additionally, we observed increases in a bone formation marker (P1NP) in the *Galnt11*-deficient mice, indicative of bone remodeling. The findings of increased trabecular bone volume and decreased cortical bone volume among *Galnt11*-deficient mice resembles what has been previously documented in individuals with renal osteodystrophy, a common complication associated with chronic kidney disease (24). Since the role of megalin in renal reabsorption of vitamin D has been elucidated, this similarity in bone phenotype associated with *Galnt11*-deficiency and chronic kidney disease hints at the possibility of a shared mechanism. The increase in trabecular bone and decrease in cortical bone documented in renal osteodystrophy has been attributed to secondary hyperparathyroidism. In this instance, low blood levels of vitamin D and calcium found in renal failure stimulate the parathyroid glands to produce consistently high levels of PTH. Chronically elevated levels of PTH result in an increase in the ratio of trabecular to cortical bone. The increased levels of PTH seen in the *Galnt11*-deficient mice likely impact the increase in trabecular and decrease in cortical bone thickness observed.

PTH levels were notably and consistently elevated in *Galnt11*-deficient mice relative to *WT*. While the observed megalin-mediated decrease in vitamin D likely leads to decreased intestinal calcium absorption and secondary hyperparathyroidism, other potential Galnt11-dependent and independent effects cannot be excluded. However, it is known that vitamin D normally decreases PTH production and parathyroid gland proliferation (27), thus the effects of a sustained decrease in vitamin D levels may further act to increase PTH levels in this mouse model. Additionally, megalin is known to play a role in the renal catabolism of PTH (25). Thus, the combined effects of decreased calcium, decreased vitamin D, and decreased renal catabolism of PTH may be responsible for the notable increases in PTH observed in *Galnt11*-deficient animals.

Reduced calcium and increased PTH are expected to increase the expression of the gene encoding the 1α-hydroxylase, which converts 25-hydroxyvitamin D to 1,25-dihydroxyvitamin D in the proximal tubules of the kidney (26). Interestingly, even though there was an increase in the expression of 1α-hydroxylase gene in *Galnt11*-deficient animals, consistent with the altered calcium and PTH levels, this did not result in the normalization of 1,25-dihydroxyvitamin D levels. However, given that the primary defect in the *Galnt11-deficient* animals centers on the inefficient uptake of vitamin D by DBP within the kidney, the changes in expression of the intracellular 1α-hydroxylase within cells of the kidney may not be expected to result in significant increases in 1,25-dihydroxyvitamin D levels.

Phosphate levels remained normal upon loss of *Galnt11*. Phosphate homeostasis is regulated by Fgf23, a phosphate-regulating hormone that is produced in and secreted from bone osteocytes. The bone responds to serum phosphate levels by inducing the expression of another member of the Galnt family, Galnt3, which glycosylates Fgf23, protecting it from inactivating cleavage (28). Intact, active Fgf23 then modulates phosphate reabsorption in the kidney. We observed no significant differences in cleaved (inactive) or intact (active) Fgf23 between *WT* and *Galnt11*-deficient animals, suggesting that the loss of Galnt11 is not affecting Fgf23 and that Fgf23 is acting to maintain appropriate phosphate levels in this model.

It is a widely recognized phenomenon that some patients require high doses of vitamin D to maintain normal vitamin D levels (29). This is usually ascribed to gastrointestinal malabsorption of vitamin D, even in patients in whom there is no evidence of malabsorption. Our data suggest the possibility that there may be variants in *GALNT11* that lead to renal losses of vitamin D through impaired DBP reabsorption, and such variants could create a clinical need for high doses of vitamin D to maintain normal levels in affected individuals. Further, differences in vitamin D homeostasis due to genetic variants in *GALNT11* may in part explain why some, but not all patients, appear to benefit from vitamin D supplementation (30, 31).

In summary, we provide evidence for the direct role of Galnt11 in vitamin D reabsorption, impacting mineral homeostasis and bone composition (Fig. 5). Moreover, we demonstrate effects on the bones of *Galnt11*-deficient animals that are consistent with secondary hyperparathyroidism. These results highlight a previously unappreciated role for Galnt11 in vitamin D and mineral homeostasis and the integrity of the skeletal system.

## Experimental procedures

### Animal breeding and genotyping

*Galnt11*-deficient mice were backcrossed into the C57BL/6NHsd inbred mouse background for at least six generations before analysis. Heterozygous *Galnt11* animals (*Galnt11*^*+/−*^) were crossed to generate *WT* and homozygous *Galnt11*-deficient (*Galnt11*^*−/−*^) siblings to be used for all experiments. Genomic DNA was extracted from mouse ear punches using the DNeasy Blood & Tissue Kit (Qiagen #69506) and PCR was performed as previously described (19). Experimental procedures were reviewed and approved by the Animal Care and Use Committee of the National Institutes of Health (ASP #19-1016).

### Quantitative analysis of femurs using micro-CT

Femur bones of *Galnt11*^*−/−*^ and WT littermate mice underwent micro-CT analysis. Dissected femurs were preserved in paraformaldehyde prior to the analysis and wrapped in Parafilm during scanning to prevent drying. Femurs were scanned in a μCT 50 scanner (Scanco Medical) at 70 kVp, 76 μA, 0.5 Al filter, 300 ms integration time, and 10 μm voxel dimension. Analyze (version 14.0; AnalyzeDirect) was used to reconstruct and analyze images. Reconstructed images were calibrated to a linear standard curve using five hydroxyapatite (HA) phantoms with known densities (mg/cm^3^). Both trabecular and cortical volumes of interest (VOI) were defined for each femur according to standard algorithms (32, 33). In brief, the growth plate cross-section served as an anatomical reference from which the long axis of the VOI was defined. The trabecular VOI was defined to begin at 67% of the proximal-to-distal length of the femur and extend distally to 0.6 mm proximal from the growth plate. The cortical VOI was defined to start at 56% of the proximal-to-distal length of the femur and extend 1 mm distally toward the growth plate. The minimum threshold for trabecular bone was defined as 300 (mg HA/cm^3^) and the minimum threshold for cortical bone was defined as 550 (mg HA/cm^3^). Morphometrics and densities were then calculated for trabecular and cortical regions.

### Blood chemistry analysis

Blood was collected, and plasma or serum was isolated. Blood calcium, phosphorous, vitamin D, and PTH were analyzed by standard laboratory methods according to manufacturer’s descriptions. Calcium (cat. #ab102505) and phosphate (cat. #ab65622) colorimetric assay kits were purchased from abcam. 1,25-dihydroxyvitamin D ELISA assay kit was purchased from Immunodiagnostic Systems Inc (cat. #AC-62F1). Mouse/Rat 25-OH Vitamin D ELISA assay kit was purchased from Eagle Biosciences (cat. #VID21-K01). Mouse PTH 1-84 ELISA kit was purchased from Quidel (cat. #602305, #60-6300). Intact Fgf23 ELISA assay was purchased from Kainos (cat. #CY-4000). TRAP5b ELISA and P1NP EIA assays were purchased from Immunodiagnostic Systems (cat. #SB-TR103, #AC-33F1).

### Quantitative PCR

Kidney tissues from mice were used for total RNA extraction and 1 μg of total RNA was reverse transcribed (Bio-Rad, Hercules). Quantitative real-time RT-PCR analyses were carried out using the following primer pair: For *Cyp27b1*, forward 5′-GGGCCAATATGGTCTGGCAG-3′ and reverse 5′-GGACAGTGACTTTCTTGTCGC-3′. Fluorescently labeled SYBR Green dye (Roche Applied Science) in a QuantStudio 7 (Thermo Fisher Scientific, Applied Biosystems). Relative mRNA quantification was calculated by delta-delta Ct method and Ct values were normalized with 29S as an internal control.

### Statistical analysis

For blood chemistry analysis and gene expression study, Student's two-tailed non-paired *t* test was used and *p* values of <0.05 were regarded as statistically significant. Data from the micro-CT analysis, comparing the *Galnt11*^+/+^ and *Galnt11*^*−/−*^ mice, were analyzed using an unpaired parametric *t* test with Welch’s correction (Prism version 8.4.1; GraphPad Software), where *p* < 0.05 was considered statistically significant. Significance is designated with asterisks (∗*p* < 0.05, ∗∗*p* < 0.01, ∗∗∗*p* < 0.001). Means ± SD are shown in graphs.

## Data availability

All data related to this study are contained within the manuscript.

## Supporting information

This article contains supporting information.

## Conflict of interest

The authors declare that they have no conflicts of interest with the contents of this article.
