# Supplementary material for: Loss of the glycosyltransferase Galnt11 affects vitamin D homeostasis and bone composition
Source: J Biol Chem. 2024 Mar 12;300(4):107164. doi: 10.1016/j.jbc.2024.107164 (PMC11001633; doi:10.1016/j.jbc.2024.107164)
Supplement: Supporting Figures S1 and S2 [file mmc1.pdf]

Supporting Information

Loss of the glycosyltransferase Galnt11 affects vitamin D homeostasis and bone composition

E Tian[1], Caroline Rothermel[1+], Zachary Michel[2], Luis Fernandez de Castro[2], Jeeyoung Lee[1], Tina Kilts[1], Tristan Kent[2], Michael T. Collins[2], and Kelly G. Ten Hagen[1] *

From the [1]Developmental Glycobiology Section, National Institute of Dental and Craniofacial Research, National Institutes of Health, Bethesda, Maryland 20892-4370
[2]Skeletal Disorders and Mineral Homeostasis Section, National Institute of Dental and Craniofacial Research, National Institutes of Health; [+]currently at the University of California, Irvine School of Medicine

Running title: *Galnt11 influences vitamin D homeostasis*

This File includes:

Figure S1
Figure S2

Figure S1

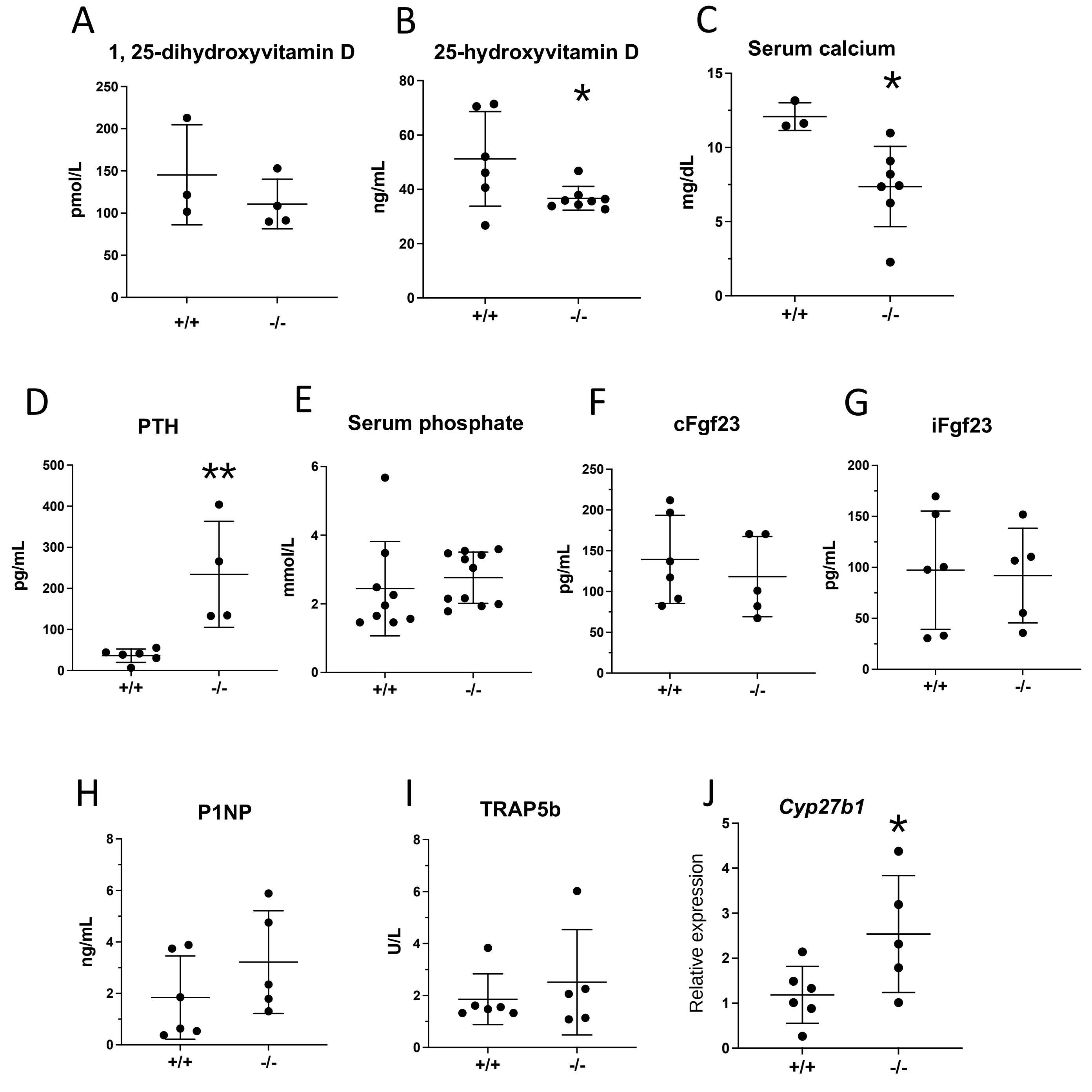

Figure S1. Circulating bone turnover markers in female *WT* and *Galnt11*-deficient mice. Data for female *WT* (+/+) and *Galnt11*-deficient mice (-/-) is shown for circulating (A) 1,25-dihydroxyvitamin D; (B) 25-hydroxyvitamin D; (C) calcium; (D) PTH; (E) phosphate; (F) cleaved Fgf23 (cFgf23); (G) intact Fgf23 (iFgf23); (H) P1NP; and (I) TRAP5b. (J) QPCR analysis of *Cyp27b1* gene expression in female *WT* (+/+) and *Galnt11*-deficient mice (-/-). *, $p < 0.05$; **, $p<0.01$.

Figure S2

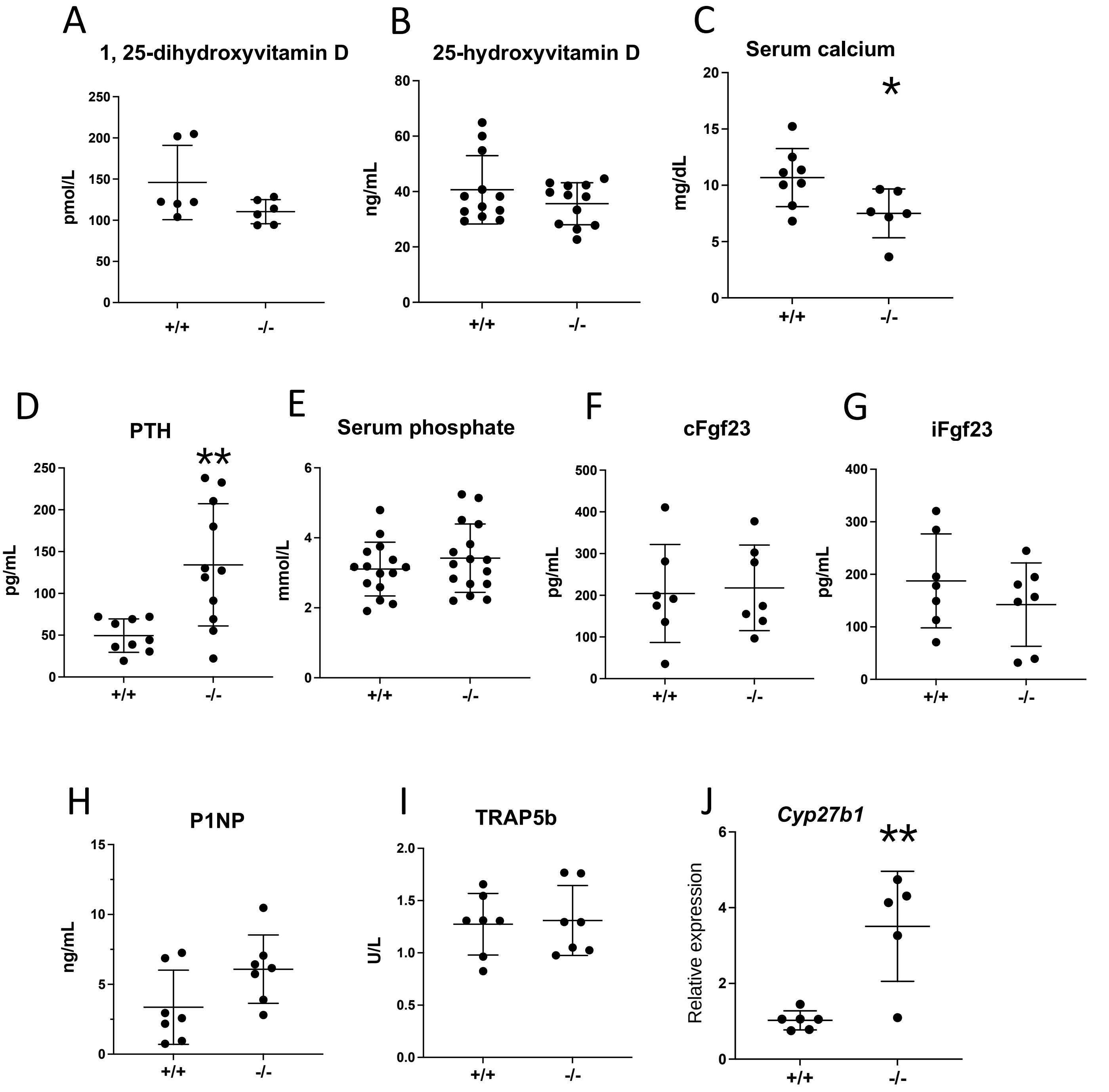

Figure S2. Circulating bone turnover markers in male *WT* and *Galnt11*-deficient mice. Data for male *WT* (+/+) and *Galnt11*-deficient mice (-/-) is shown for circulating (A) 1,25-dihydroxyvitamin D; (B) 25-hydroxyvitamin D; (C) calcium; (D) PTH; (E) phosphate; (F) cleaved Fgf23 (cFgf23); (G) intact Fgf23 (iFgf23); (H) P1NP; and (I) TRAP5b. (J) QPCR analysis of *Cyp27b1* gene expression in male *WT* (+/+) and *Galnt11*-deficient mice (-/-). *, $p < 0.05$; **, $p<0.01$.
